# Poorly controlled seizures are associated with severe neuropathological changes in dementia

**DOI:** 10.1002/alz.088243

**Published:** 2025-01-09

**Authors:** Ifrah Zawar, Mark S Quigg, Carol A Manning, jaideep kapur

**Affiliations:** ^1^ University of Virginia, Charlottesville, VA USA

## Abstract

**Background:**

Seizures maybe associated with worse neuropathology findings in people with dementia. However, the role of seizure control and how it may impact post‐mortem histopathology findings in people with dementia remains unexplored.

**Method:**

We used the longitudinal, multicenter National Alzheimer Coordinating Center data from 9/2005 to 12/2021 to evaluate the association between seizure control and histopathological neurodegenerative changes in people with dementia. The differences between baseline demographics, cognition, mortality and neuropathology findings were investigation among three groups: 1) Recurrent seizures (over the preceding 12 months), 2) Remote seizures (history of seizures but no seizures over the preceding 12 months) and 3) No history of seizures (controls). Pearson’s Chi‐squared test, fisher’s exact test and Kruskal‐Wallis Rank sum tests were utilized for analysis as deemed appropriate.

**Result:**

Of 10,474 NACC participants who died, mortality was higher (p<0.001) among recurrent seizure (N=407, 56%) patients compared to remote (N=221, 35%) seizures and controls (N=9846,34%). Among 6085 who underwent autopsy, 294 had recurrent seizures, 151 had remote seizures and 5640 had no seizures. Recurrent seizure patients were younger than controls at death (76 vs 82 years, p<0.001). There were no group differences in education, sex, race and ethnicity. However, recurrent seizure patients had worse cognition compared to other groups. Lobar atrophy, vascular pathology, atherosclerosis of circle of willis and substantia nigra hypopigmentation were comparable among the 3 groups. However, recurrent seizure patients had more cerebral cortex atrophy (p<0.001), hippocampal atrophy (p=0.016) and locus ceruleus hypopigmentation (p=0.011). While Lewy body pathology was similarly prevalent among the groups, frontotemporal degeneration with tau pathology was less prevalent (p=0.013) among the recurrent seizures compared to other groups. Recurrent seizure patients had evidence of more severe Alzheimer’s disease pathology (AD) including thal phase for amyloid, Braak stage for neurofibrillary degeneration and density of neocortical neuritic plaque (p<0.001).

**Conclusion:**

Our findings suggest that recurrent seizures compared to remote seizures prior to death are associated with postmortem evidence of more severe AD pathology and cortical and hippocampal atrophy. These findings emphasize the need for more aggressive seizure control to mitigate the potential impact of seizures on neurodegeneration.

